# Effects of Danshen tablets on pharmacokinetics of atorvastatin calcium in rats and its potential mechanism

**DOI:** 10.1080/13880209.2018.1424209

**Published:** 2018-01-11

**Authors:** Sen Sun, Rong Wang, Jie Fan, Guoqing Zhang, Hai Zhang

**Affiliations:** a Department of Pharmacy, Shanghai Eastern Hepatobiliary Surgery Hospital, Shanghai, China;; b Department of Pharmacy, Shanghai 9th People's Hospital, Shanghai Jiao Tong University School of Medicine, Shanghai, China;; c Department of Pharmacy, Shanghai First Maternity and Infant Hospital, Tongji University School of Medicine, Shanghai, China

**Keywords:** Herb-drug interaction, LC-MS/MS, metabolism

## Abstract

**Context:** Danshen tablets (DST), an effective traditional Chinese multi-herbal formula, are often combined with atorvastatin calcium (AC) for treating coronary heart disease in the clinic.

**Objective:** This study investigated the effects of DST on the pharmacokinetics of AC and the potential mechanism.

**Materials and methods:** The pharmacokinetics of AC (1 mg/kg) with or without pretreatment of DST (100 mg/kg) were investigated using LC-MS/MS. The effects of DST (50 μg/mL) on the metabolic stability of AC were also investigated using rat liver microsome incubation systems.

**Results:** The results indicated that *C*_max_ (23.87 ± 4.27 vs. 38.94 ± 5.32 ng/mL), AUC_(0–_*_t_*_)_ (41.01 ± 11.32 vs. 77.28 ± 12.92 ng h/mL), and *t*_1/2_ (1.91 ± 0.18 vs. 2.74 ± 0.23 h) decreased significantly (*p* < 0.05) when DST and AC were co-administered, which suggested that DST might influence the pharmacokinetic behavior of AC when they are co-administered. The metabolic stability (*t*_*1/2*_) of AC was also decreased (25.7 ± 5.2 vs. 42.5 ± 6.1) with the pretreatment of DST.

**Discussion and conclusions:** This study indicated that the main components in DST could accelerate the metabolism of AC in rat liver microsomes and change the pharmacokinetic behaviors of AC. So these results showed that the herb-drug interaction between DST and AC might occur when they were co-administered. Therefore, the clinical dose of AC should be adjusted when DST and AC are co-administered.

## Introduction

Atorvastatin, a synthetic and lipophilic statin, inhibits 3-hydroxy-3-methylglutaryl coenzyme A (HMG-CoA) reductase, the rate-limiting enzyme in cholesterol biosynthesis (Dybro et al. 2016; Eng et al. 2016; Cruz-Correa et al. 2017; Dennison et al. 2017). Atorvastatin is commonly prescribed for the treatment of hypercholesterolemia and for the prevention of coronary heart disease (Kadam et al. 2016; Liu et al. 2016; Bautista et al. 2017; Kasabri et al. 2017; Kashihara et al. 2017). Growing experimental evidence shows the cholesterol-independent pleiotropic effects of this drug, including protection against Alzheimer’s disease, Parkinson’s disease, multiple sclerosis, depression, convulsion, peripheral neuropathy and intracerebral hemorrhage (Roth et al. 2016; Shu et al. 2016; Wang et al. 2016). Most of the statins, except pravastatin, are metabolized by the cytochrome P450 (CYP) enzymes (Wei and Zhang 2015; Hsiao et al. 2016). Interactions involving CYP are therefore possible. For example, itraconazole has been shown to increase the area under the plasma concentration-time curve (AUC) of atorvastatin acid and lactone by three- and four-fold, respectively (Higgins et al. 2014; Dybro et al. 2016).

Danshen tablets (DST), an effective traditional Chinese multi-herbal formula, are widely used in treating cardiovascular diseases (Shi et al. 2016; Yin et al. 2016; Zhang et al. 2016; Yao et al. 2017). As we know, atorvastatin calcium (AC) and DST are often simultaneously used for treating coronary heart disease in Chinese clinics. However, it is unknown whether there is an interaction between AC and DST. A better understanding of the pharmacokinetic interaction between DST and AC would help link data from pharmacological assays to clinical effects, thus facilitating the design of rational dosage regimens and avoiding the occurrence of adverse reactions (Yang et al. 2011).

This study investigates the effects of DST on the pharmacokinetics of AC in rats, and to clarify the herb-drug interaction potential between DST and AC.

## Materials and methods

### Materials and reagents

Standards of AC (purity >98%) and simvastatin (purity >98%) was purchased from the National Institute for the Control of Pharmaceutical and Biological Products (Beijing, China). d-Glucose-6-phosphate, glucose-6-phosphate dehydrogenase and NADP^+^ were obtained from Sigma Chemical Co. (St. Louis, MO, USA). Pooled RLM were purchased from BD Biosciences Discovery Labware (Woburn, MA, USA). All other reagents and solvents were of analytical reagent grade. DST tablets were purchased from Guangdong Baiyunshan Pharmaceutical Co., Ltd (Guangzhou, China). Acetonitrile and methanol were purchased from Fisher Scientific (Fair Lawn, NJ, USA). Formic acid was purchased from Anaqua Chemicals Supply Inc. Limited (Houston, TX, USA). Ultrapure water was prepared with a Milli-Q water purification system (Millipore, Billerica, MA, USA). All other chemicals were of analytical grade or better.

### Animals

This animal experimental protocol was approved by the Animal Ethics Committee of the Second Military Medical University. Male Sprague–Dawley rats weighing 220–250 g were supplied by Sino–British Sippr/BK Lab Animal Ltd (Shanghai, China). The rats were maintained in an air-conditioned animal quarter at 22 ± 2 °C and 50 ± 10% relative humidity. Water and food (Laboratory Rodent Chow, Shanghai, China) were allowed *ad libitum*. The animals were acclimatized to the facilities for five days, and then fasted with free access to water for 12 h prior to each experiment.

### Instrumentation and conditions

The analysis was performed on an Agilent 1290 series liquid chromatography system (Agilent Technologies, Palo Alto, CA, USA) that included a binary pump, an on-line vacuum degasser, a surveyor autosampling system, a column temperature controller and an Agilent 6460 triple-quadrupole mass spectrometer (Agilent Technologies, CA, USA) with Turbo Ion spray, which is connected to the liquid chromatography system. The Agilent MassHunter B 4.0 (Agilent Technologies, CA, USA) software was used to control the equipment and for data acquisition. The chromatographic analysis of AC was performed on a Waters X-Bridge C18 column (Milford, MA, USA) (3.0 × 100 mm, i.d.; 3.5 μm) at room temperature. The mobile phase was water (containing 0.1% formic acid) and acetonitrile (30:70, v:v) at a flow rate of 0.4 mL/min.

The mass scan mode was the positive MRM mode. The precursor ion and product ion were *m/z* 559.2 → 440.2 for AC and *m/z* 419.9 → 199.4 for the simvastatin, respectively. The collision energy for AC and simvastatin were 30 and 25 eV, respectively. The MS/MS conditions were optimized as follows: fragmentor, 140 V; capillary voltage, 4 kV; nozzle voltage, 500 V; nebulizer gas pressure (N_2_), 40 psig; drying gas flow (N_2_), 10 L/min; gas temperature, 350 °C; sheath gas temperature, 400 °C; and sheath gas flow, 11 L/min.

### Preparation of standard solutions

The stock solution of AC and simvastatin were prepared in methanol at a concentration of 2.00 and 1.00 mg/mL, respectively. The stock solutions of atorvastatin were further diluted with methanol to give a series of standard solution in the range of 5–2500 ng/mL, from which the spiked plasma samples were prepared by appropriate dilution. Quality control (QC) samples were prepared at low (2 ng/mL), medium (50 ng/mL) and high (400 ng/mL) concentrations in the same way as the plasma samples for calibration.

### Preparation of rat plasma samples

Each plasma sample (100 μL) was spiked with 10 μL of the simvastatin (0.5 μg/mL). The mixture was then extracted with 190 μL of acetonitrile by vortexing for 1 min, and centrifuged at 12,000 rpm for 10 min. The supernatant was removed into an injection vial, and a 3 μL aliquot was injected into the LC-MS/MS system for analysis.

### Validation of LC-MS/MS method

The method validation assays were performed according to the United States Food and Drug Administration (FDA) guidelines.

### Specificity

Specificity was investigated by analyzing six individual blank rat plasma samples, which were compared to those obtained by the spiking analyte and IS into the corresponding blank plasma sample to monitor interference.

### Linearity and sensitivity

For the calibration curve, nine concentrations of calibration standards (1, 2, 5, 10, 20, 50, 100, 200 and 500 ng/mL) were processed and determined as described above. The calibration curves for AC were constructed by plotting peak area ratios of the analyte to simvastatin against plasma concentrations. The lower limit of quantification (LLOQ) was determined as the concentration of the analyte with a signal-to-noise ratio of 10.

### Precision and accuracy

To determine intra-day precision and accuracy, six replicates of QC samples at low, medium and high concentration levels (2, 50, and 400 ng/mL) were prepared and analyzed on the same day. Inter-day precision and accuracy were evaluated on three independent days. The intra- and inter-day precisions were expressed as the relative standard deviation (RSD) value and the accuracy as the RE value.

### Extraction recovery and matrix effect

The extraction recovery was determined by calculating the ratio of QC (2, 50, and 400 ng/mL) samples obtained against those originally spiked in the blank plasma; this step was replicated six times. The matrix effect was evaluated by comparing the solution spiked with the blank processed matrix with the solution at three different QC concentrations; this step was replicated six times. The extraction recovery and matrix effect of the IS were also determined.

### Stability

The short-term stability was evaluated by determining QC samples at room temperature for 12 h. The auto-sampler stability was detected in auto-sampler after preparation for 24 h. The freeze-thaw stability was determined through three freeze-thaw cycles on consecutive days. The long-term stability was assessed by storing the QC samples at −40 °C for 15 days.

### Pharmacokinetic study

For pharmacokinetic study *in vivo*, 12 rats were equally randomized to two groups, six rats in each group, including AC-only group (1 mg/kg) (A) and AC (1 mg/kg) + DST (100 mg/kg) group (B). The AC and DST fraction powders were all homogenized in water solution with a mortar and pestle. Blood samples (0.25 mL) were collected into a heparinized tube via the oculi chorioideae vein before drug administration and at 0.083, 0.25, 0.5, 1, 2, 3, 4, 6, 8, 12 and 24 h after drug administration. After centrifuging at 3500 rpm for 10 min, the supernatant was obtained and frozen at −40 °C until analysis.

### Pharmacokinetic analysis

Plasma data was subjected to non-compartmental pharmacokinetic analysis by using DAS version 3.0 (BioGuider Co., Shanghai, China), and parameters were calculated. The maximum plasma concentration (*C*_max_) and time to *C*_max_ (*T*_max_) were obtained directly from the concentration-time curves. The elimination rate constant (*k*) was calculated by linear regression of the final linear part of plasma concentration against time. The elimination half-life (*t*_1/2_) was calculated from the formula *t*_1/2_ *=* 0.693/*k*. The area under the concentration-time curve (AUC_0–_*_t_*) was calculated by using the linear trapezoidal rule. The mean residence time (MRT) was calculated as AUMC_0–_*_∞_*/AUC_0–_*_∞_*, whereby AUMC_0–_*_∞_*represented the area under the first moment plasma concentration-time curve. All data were presented as mean ± SD.

### Inhibitory effects of DST on the metabolic stability of AC in rat liver microsomes

Rat liver microsomes were used to determine the metabolic rate of AC. The assay conditions and reaction mixtures were similar as reported previously (Qi et al. 2013; Qin et al. 2014). The reaction mixture was incubated at 37 °C for 5 min and then AC (100 μM) was added. The effects of DST on the metabolic rate of AC was investigated by adding 50 μg/mL of DST to rat liver microsomes and preincubating for 30 min at 37 °C, and then AC (100 μM) was added. Aliquots of 30 μL were collected from reaction volumes at 0, 1, 3, 5, 15, 30 and 60 min and 60 μL ice-cold acetonitrile was added to terminate the reaction, and then the sample preparation method was the same as the plasma sample preparation method and determined by LC-MS/MS.

The half-life (*t*_1/2_) *in vitro* was obtained using equation: *t*_1/2_ *=* 0.693/*k*.

### Statistical analysis

Experimental values are expressed as mean ± SD. Statistical analysis of results obtained from clinical study was performed using Student’s paired *t-*test. Differences were considered statistically significant when *p* values were <0.05. Statistical analysis was conducted using Graph-Pad Prism version 3.0 for Windows (San Diego, CA, USA).

## Results

### Sample preparation

Due to the heterogeneous nature of plasma, a sample pretreatment procedure is often needed to remove protein and potential interfering substances before the LC-MS/MS analysis. Currently, the most widely used biological sample preparation methods are protein precipitation, solid phase extraction and liquid-liquid extraction. All of these sample pretreatment methods were investigated to achieve good resolution and high recovery of the analyte from spiked biological matrices. Finally, protein precipitation was selected for AC. The highest recovery was obtained using acetonitrile as a protein precipitant.

### Method validation

#### Selectivity

**Figure 1.**
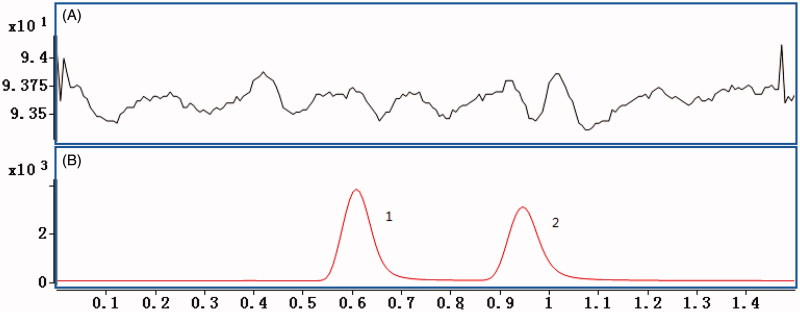
(A) Representative chromatograms of blank plasma; (B) blank plasma spiked with AC (1) and simvastatin (2).

In the present study, the selectivity was examined using independent plasma samples from six different rats. As shown in Figure 1, no obvious interference was observed in the representative chromatogram of a blank plasma sample at the retention times of the analyte and IS.

### Linearity and lower limit of quantification

Linearity for AC was obtained over the concentration range of 1–500 ng/mL. A typical calibration curve was *y* = 0.0157*x* + 0.00312 (*r*^2^= 0.998), where *y* represents the peak area ratios of AC to the IS and *x* represents the plasma concentrations of AC. The LLOQ of AC in rat plasma was 1 ng/mL.

### Precision and accuracy

The precision and accuracy data for the plasma samples are presented in Table 1. The intra- and inter-day precision values (RSD) were less than 10%, and the accuracy (RE) ranged from −6.89 to 8.00%. These data indicated that the accuracy and precision of the method were satisfactory.

**Table 1.** The intra-day and inter-day precision and accuracy of AC in plasma samples (*n* = 6).

	Intra-day			Inter-day		
Spiked concentration (ng/mL)	Concentration measured (ng/mL)	Precision (%, RSD)	Accuracy (%, RE)	Concentration measured (ng/mL)	Precision (%, RSD)	Accuracy (%, RE)
2	1.89	5.35	−5.50	2.16	6.18	8.00
50	43.46	6.84	6.92	54.32	5.62	8.64
400	422.51	7.28	5.63	372.46	6.67	−6.89

### Extraction recovery and matrix effects

The matrix effects were examined to assess the possibility of ion suppression or enhancement. The matrix effects ranged from 91.25 to 95.68% for AC over the three levels of QC samples. The results indicated that no obvious matrix effects were present. The overall mean recoveries of AC in plasma at the three different concentration levels were found to be 87.65–93.51% with a RSD less than 10%, which indicated that the extraction procedure was consistent and reproducible.

#### Stability

The results of the short-term stability, auto-sampler stability, freeze and thaw stability and long-term stability are shown in Table 2. It was demonstrated that the stability offered by this method was satisfactory, with the REs and RSD for all samples within the general assay acceptability criteria. These results showed that the samples were sufficiently stable to allow for routine analysis as part of the pharmacokinetic study of AC.

**Table 2.** Stability of AC in plasma samples (*n* = 3).

Stability conditions	Spiked concentration (ng/mL)	Measured concentration (ng/mL)	Precision (RSD, %)	Accuracy (RE, %)
Short-term (room temperature for 12 h)	2	1.86	5.38	−7.00
	50	45.35	7.05	−9.30
	400	364.25	6.86	−8.94
Autosampler (24 h)	2	2.16	7.35	8.00
	50	53.82	5.38	7.64
	400	438.35	7.43	9.59
Three freeze/thaw cycles (−40 °C)	2	2.15	6.98	7.50
	50	53.42	8.16	6.84
	400	366.58	7.06	−8.36
Long-term (−40 °C for 15 days)	2	2.19	5.54	9.50
	50	45.35	8.16	−9.30
	400	433.25	7.55	8.31

### Pharmacokinetic study in vivo

**Figure 2.**
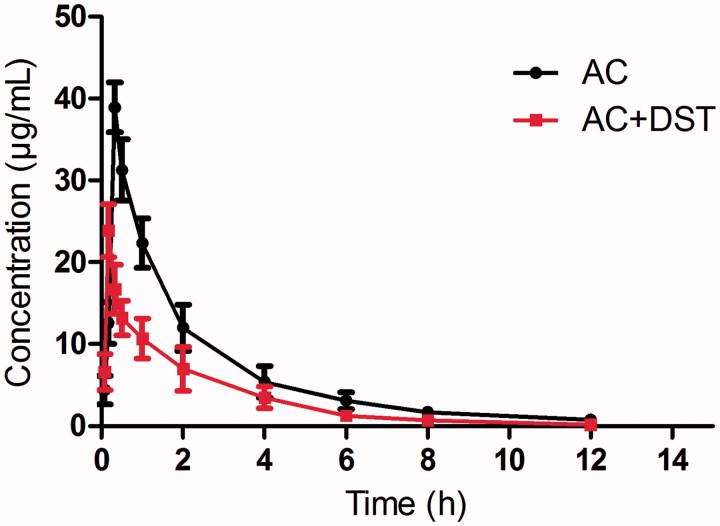
The mean concentration-time curves in rat plasma after oral administration of single AC or both AC and DST.

The analytical procedures described were used to quantify AC in rat plasma samples obtained from 12 male Sprague–Dawley rats, of which six were orally administered AC aqueous solution and the other six were orally administered AC and DST aqueous solution. The mean concentration-time curve of AC in the two treatments is shown in Figure 2, and all the pharmacokinetic parameters are shown in Table 3. The results indicated that the *C*_max_ (23.87 ± 4.27 vs. 38.94 ± 5.32 ng/mL), AUC_(0–_*_t_*_)_ (41.01 ± 11.32 vs. 77.28 ± 12.92 ng h/mL), and *t*_1/2_ (1.91 ± 0.18 vs. 2.74 ± 0.23 h) decreased significantly when DST and AC were co-administered, which suggested that DST might influence the pharmacokinetic behavior when they are co-administered. These results suggested that the herb-drug interaction between AC and DST might occur when they are co-administered. As the plasma concentration of AC decreased when co-administered with DST, which suggested that the pharmacological activities of AC might be weakened, and therefore, the clinical dose of AC should be adjusted when they are used simultaneously. Salvianolic acid B is a major component isolated from DST, and previous studies (Wang et al. 2016) have reported that salvianolic acid B could induce the activity of CYP3A4 in a concentration-dependent manner. As AC is a substrate of CYP3A4 enzymes, which is predominantly metabolized by CYP3A4 (Lee et al. 2015), and therefore, we infer that the herb-drug interaction between AC and DST may occur due to the effects of salvianolic acid B on the activity of CYP3A4.

**Table 3.** Pharmacokinetic parameters of AC in male Sprague − Dawley rats following oral administration of AC alone or both AC and DST.

	AC	
Parameter	AC	AC + DST
*T*_max_ (h)	0.32 ± 0.03	0.18 ± 0.02*
*C*_max_ (ng mL^−1^)	38.94 ± 5.32	23.87 ± 4.27*
*t*_1/2_ (h)	2.74 ± 0.23	1.91 ± 0.18*
AUC_(0–_*_*t*_*_)_ (ng h mL^−1^)	77.28 ± 12.92	41.01 ± 11.32*
CL (L h^−1^kg^−1^)	12.91 ± 2.79	25.65 ± 6.63*
MRT (h)	2.55 ± 0.24	2.29 ± 0.19*

* *p* < 0.05 indicate significant differences from the control.

### Inhibitory effects of DST on the metabolic stability of AC in rat liver microsomes

As we know, the metabolism of AC was mainly modulated by CYP3A4 enzymes, and therefore, in this research, the effects of DST on the metabolic stability of AC were further investigated in rat liver microsomes *in vitro*. The metabolic stability of AC was 42.5 ± 6.1 min, while the metabolic stability was decreased (25.7 ± 5.2 min) in the presence of DST. The results indicated that the main components in DST could accelerate the metabolism of AC in rat liver microsomes and change the pharmacokinetic behaviors of AC. Therefore, we infer that DST could also affect the pharmacokinetic profiles of other statins (simvastatin, lovastatin) as they were also metabolized by the CYP3A4 enzymes, and herb-drug interactions should also occur when they are co-administered.

## Conclusions

In conclusion, the results indicated that DST could influence the pharmacokinetic behavior of AC when they are co-administered. The main ingredients in DST could accelerate the metabolism of AC in rat liver microsomes, and the metabolic stability of AC was decreased, which may be one of the reasons resulting in pharmacokinetic interactions when they are co-administered. Therefore, the clinical dose of AC should be adjusted when DST and AC are co-administered.
